# Comparative study of choline alfoscerate as a combination therapy with donepezil: A mixed double-blind randomized controlled and open-label observation trial

**DOI:** 10.1097/MD.0000000000038067

**Published:** 2024-06-14

**Authors:** Wankiun Lee, Manho Kim

**Affiliations:** a Department of Neurology, Chungnam National University Hospital, Daejeon, Korea; b Department of Neurology, Seoul National University Hospital, Seoul, Korea; c Department of Neurology, Neuroscience and Dementia Research Center, Seoul National University College of Medicine, Seoul, Korea; d Convergence Research Center for Dementia, Seoul National University Medical Research Center, Seoul, Korea.

**Keywords:** choline alfoscerate, cognition, donepezil

## Abstract

**Background:**

Choline alfoscerate (alpha-glycerylphosphorylcholine) is a phospholipid that includes choline, which increases the release of acetylcholine. The ASCOMALVA trial, a combination of donepezil and choline alfoscerate, slowed cognitive decline in Alzheimer disease. This study aims to replicate the effect by combining donepezil with other nootropics currently used in South Korea.

**Methods:**

The 119 patients with cognitive decline who were eligible to use donepezil, with an mini-mental state examination (MMSE) score of 26 or less, were assigned to: donepezil alone (DO); donepezil and choline alfoscerate (DN); donepezil and acetyl-l-carnitine (DA); or donepezil and ginkgo biloba extract (DG). Cognitive evaluations such as MMSE, clinical dementia rating, Alzheimer disease assessment scale-cognitive subscale (ADAS-Cog), and Alzheimer disease assessment scale-noncognitive subscale were performed at the 12th and 24th weeks from the baseline time point.

**Results:**

At the 12th week, the MMSE score increased 3.52% in the DN group, whereas it increased by 1.36% in the DO group. In the DA + DG group, it decreased by 2.17%. At the 24th week, the MMSE score showed an increase of 1.07% in the DO group and 1.61% in the DN group, but decreased by 5.71% in the DA + DG group. ADAS-Cog decreased by 0.9% in the DO group, while it improved by 13.9% in the DN group at the 12th week. At the 24th week, ADAS-Cog showed improvement in the DN group by 18.5%, whereas it improved by 9.4% in the DO group. Alzheimer disease assessment scale-noncognitive subscale also revealed better performance in the DN group than in the DO group at the 12th and 24th weeks.

**Conclusion:**

Choline alfoscerate exhibits additional cognitive improvement in both cognitive and noncognitive domains, supporting the findings of the ASCOMALVA trial.

## 1. Introduction

The elderly population constituted 18.5% in the year 2016 and is projected to increase to 31.4% in 2026, 49.8% in 2036, 66.0% in 2046, and 75.4% in 2056. Dementia is also emerging as the most urgent problem to address in the medical community as well as in society as a whole.

At the time of this study (2019), there are 5 drugs approved for sale on the market for the improvement of cognitive function: donepezil, rivastigmine, galantamine, memantine, and aducanumab. The former 3 drugs have been utilized for symptomatic cognitive improvement by inhibiting the degradation of acetylcholine. Memantine protects neurons from neurotoxicity caused by N-methyl-d-aspartate. Donepezil is the most widely used drug, with relatively fewer adverse effects than the others.

Choline alfoscerate, acetyl-l-carnitine, oxiracetam, or ginkgo biloba extract have been utilized as nootropics. Proposed mechanisms include enhancing microcirculation, serving as acetylcholine precursors, or promoting neuronal membrane regeneration.^[1–3]^

Choline serves as a precursor of acetylcholine. The degeneration of cholinergic neurons results in a decrease in the synthesis and secretion of acetylcholine, along with reduced choline acetyltransferase and acetylcholinesterase activity in Alzheimer disease.^[4,5]^ Reduced cholinergic function forms the basis for choline supplementation therapy. However, clinical trials using choline and phosphatidylcholine (lecithin) did not confirm increased synthesis and secretion of acetylcholine.^[6,7]^

Choline alfoscerate, unlike choline or lecithin, is delivered to the brain through the blood–brain barrier, leading to increased synthesis and secretion of acetylcholine.^[8,9]^ Clinical trials, including double-blind randomized placebo-controlled studies, have demonstrated its efficacy in improving memory and attention in patients with cognitive decline, such as Alzheimer disease, vascular dementia, and cerebrovascular disease.^[10,11]^

Choline alfoscerate targets the cholinergic system in Alzheimer disease pathogenesis. Alongside acetylcholinesterase inhibitors, the addition of choline alfoscerate as a strategy was proven effective by the ASCOMALVA trial. This resulted in a combination therapy that slowed cognitive decline and reduced behavioral and psychological symptoms of dementia (BPSD).^[12,13]^

The purpose of this study is to further assess whether there are additional effects in improving cognitive function by combining choline alfoscerate with donepezil.

## 2. Methods

### 2.1. Design of clinical trials

This study commenced in May 2019 as a prospective combination of double-blind randomized and open-label observation study. The subjects were consecutively recruited from outpatients who received donepezil at Seoul National University Hospital.

Then, the subjects were classified into 4 groups. The 1st group consisted of subjects taking donepezil alone (DO), and the 2nd group was designed to receive donepezil and choline alfoscerate together (DN). The 3rd group consisted of subjects taking donepezil and acetyl-l-carnitine together (DA), and the 4th group consisted of subjects taking donepezil and ginkgo biloba together (DG). Classification into either the DO or DN group was performed by random assignment, and the principal investigator and raters were unaware of this. Therefore, the clinical trial for the DO versus DN groups was a prospective double-blind randomized study. The DA and DG groups were observed in an open-labeled manner.

None of the subjects in the DO or DN group were taking acetyl-l-carnitine or ginkgo biloba extract. Subjects in the DA and DG groups were recruited if they met the relevant criteria among those who were already prescribed acetyl-l-carnitine or ginkgo biloba extract.

### 2.2. Number of subjects

A total of 200 subjects were planned to be recruited. The sample size estimation was performed using the *F* test (analysis of variance). Since there were no existing reference data for comparison between drugs, the effect size was set to 0.25. With an alpha-error set to 0.05 and power set to the recommended value of 0.8, the required number of groups was confirmed to be 4, and the required sample size was found to be 180. Considering a dropout rate of about 10%, the goal was to recruit 50 people per group with a target of 200. In the ASCOMALVA trial, the target was set at 210 people.

### 2.3. Screening

The inclusion criteria were as follows:

- Individuals aged over 50 and under 85.
- Individuals who can understand and judge the explanation on their own, or who can receive assistance from a representative.
- Individuals with Alzheimer disease according to the NINCDS-ADRDA diagnostic criteria,^[14]^ with an mini-mental state examination (MMSE) score of 26 or less.
- Individuals who have been taking donepezil stably for at least 3 months.
- Individuals who are not taking acetyl-l-carnitine and ginkgo biloba when taking donepezil alone or in combination with choline alfoscerate.
- Individuals who consented to participate in the study.

The exclusion criteria were as follows:

- Individuals exhibiting allergies or hypersensitivity to the test drug.
- Among those with clinically significant digestive system disease, individuals who refuse to take the test drug due to nausea or vomiting caused by acetylcholinesterase inhibitors.
- Individuals who have undergone surgery that may interfere with food absorption or cannot take medications orally.
- Individuals who have shown significant abnormalities in liver function or renal function during the study.
- Individuals with other major psychiatric disorders.
- Individuals with unstable physical conditions or diseases that may impede proper evaluation and treatment.
- Individuals with alcohol or drug addiction.
- Individuals participating in another clinical trial.

### 2.4. Protocol

Following the screening, basic demographic and clinical information such as family history and past medical history were collected from subjects. Baseline cognitive and noncognitive functions were measured using the Korean version of mini-mental state examination (K-MMSE), clinical dementia rating (CDR), Alzheimer disease assessment scale-cognitive subscale (ADAS-Cog), and Alzheimer disease assessment scale-noncognitive subscale (ADAS-Noncog). The Beck Depression Inventory-II was used for screening depression. Choline alfoscerate was administered as a 400 mg tablet (JW Inc, brand name Newglia), taken 3 times a day. A medication diary was provided to monitor compliance, and subjects with compliance below 80% were excluded. Cognitive function tests were performed at the 12th and 24th weeks. During this time, drug side effects and compliance were also evaluated. Patients were instructed not to take other dementia-related drugs or medications that could affect memory or cognitive function during the test period.

### 2.5. Randomization and masking

For the DO or DN group, subjects were assigned through a web-based randomization process. The enrolled study subjects were randomly assigned in a 1:1 ratio using a random number table generated by the internet program http://www.randomizer.org. A researcher not involved in the treatment and evaluation of the study subjects assigned the subjects according to the random number table. Both the investigator (or delegation log) and rater were blinded to the assignments. An independent clinical research coordinator performed randomization and provided the choline alfoscerate from the clinical research pharmacy.

### 2.6. Endpoints

The primary endpoint of this study was the percent improvement in MMSE score. From the baseline, we compared DN to the DO or DA + DG groups at the 12th and 24th weeks. The secondary endpoints were percent improvement scores of CDR, ADAS-Cog, ADAS-Noncog, and Stroop test scores at the 12th and 24th weeks.

### 2.7. Data analysis

The demographic variables and characteristics of the 4 groups were descriptively summarized. For continuous data, the mean and standard deviation were calculated, and differences among groups were confirmed using *t* tests. For categorical data, absolute values and relative frequencies were obtained, and differences among groups were assessed using the Chi-squared test or Fisher exact test. The MMSE score, the primary efficacy endpoint, was evaluated for effectiveness using the average percent change value of the scores before administration, at the 12th week, and at the 24th week. The average values of each group were assessed using paired *t* tests or repeated measures analysis of variance tests, and improvements after 12 weeks and 24 weeks were compared to the baseline. Changes in secondary efficacy endpoints were evaluated similarly. In the case of CDR, improvement was assessed and treated as a categorical variable using the yes/no method, and group comparisons were conducted.

In data analysis, outliers were identified using the interquartile range (IQR) rule. A value greater than or equal to Q3 + 1.5 * IQR or less than or equal to Q1 − 1.5 * IQR was defined as an outlier. Additionally, when analyzing the MMSE and ADAS, we attempted to define responders and nonresponders. Considering that cognitive function naturally declines over time, a decrease was classified as “nonresponder.” However, in the case of maintenance or improvement in cognitive function, participants were determined as “improvement.”

This study complied with the 2013 Helsinki Declaration and ICH-GCP to ensure the ethicality of the study. It was approved by the Institutional Review Board of Seoul National University Hospital (H-1812-112-998), and written informed consent was obtained from each participant. The study was conducted after IRB approval.

## 3. Results

### 3.1. Study subjects and demographic data

A total of 119 participants were recruited, necessitating a modification to the allocation plan despite the initial design of allocating 50 subjects for each group. Changes in domestic insurance guidelines during the research process resulted in relatively small numbers of subjects allocated to the DA and DG groups. Further enrollment of subjects up to 50 became impossible.

**Figure 1.**
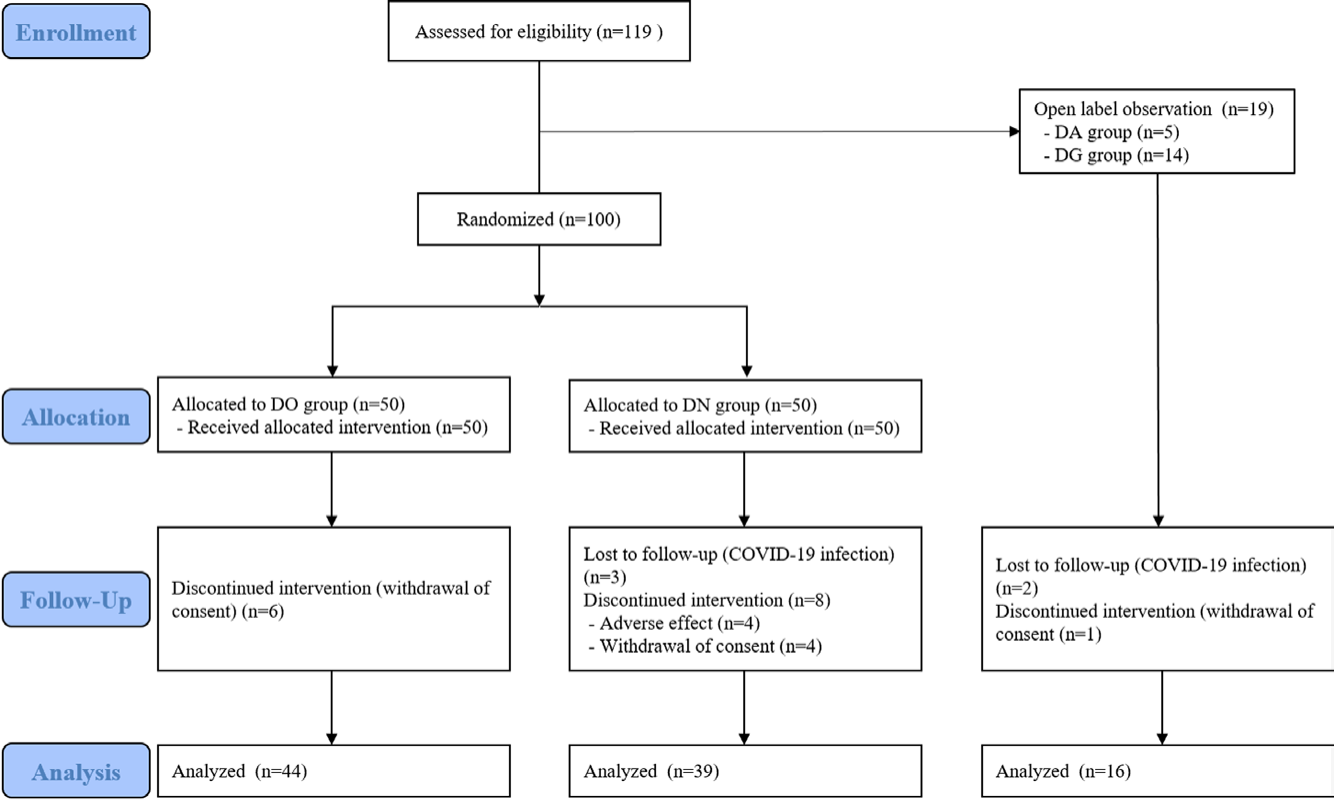
Flow chart of this study.

Thus, the DO and DN groups became the primary focus of this study. Consequently, the DO and DN groups were assigned 100 subjects (50:50), while 5 subjects were assigned to the DA group and 14 subjects to the DG group. Therefore, it was decided to refer to the DA and DG groups as another active control in consideration of the number of subjects during the analysis. All 119 subjects met the eligibility criteria and completed initial registration. Subjects in the “DA + DG group,” comprising a total of 19 subjects, were prospectively observed (open-labeled). The remaining 100 subjects were randomly assigned to either the DO or DN group in batches of 50. Six subjects dropped out from the DO group, eleven from the DN group, and 3 from the DA + DG group. Finally, 44 subjects in the DO group, 39 in the DN group, and 16 in the DA + DG group were included in the analysis (Fig. 1). The number of recruited subjects and demographic data for each group were summarized in Table 1.

**Table 1:** The number of recruited subjects and demographic data for each group.

Group categorization	Therapeutic regimen	Recruited subjects	Sex (n)		Age (mean ± SD)
DO group	Donepezil only	50	Female Male	45 5	73.8 ± 8.2
DN group	Donepezil + choline alfoscerate	50	Female Male	36 14	75.2 ± 6.6
DA group	Donepezil + acetyl-l-carnitine	5	Female Male	5 0	78.4 ± 5.1
DG group	Donepezil + ginkgo biloba extract	14	Female Male	10 4	77.4 ± 5.6

DA = administration of donepezil and acetyl-l-carnitine, DG = administration of donepezil and ginkgo biloba extract, DN = administration of donepezil and choline alfoscerate together, DO = administration of donepezil alone, SD = standard deviation.

### 3.2. Primary endpoint

**Figure 2.**
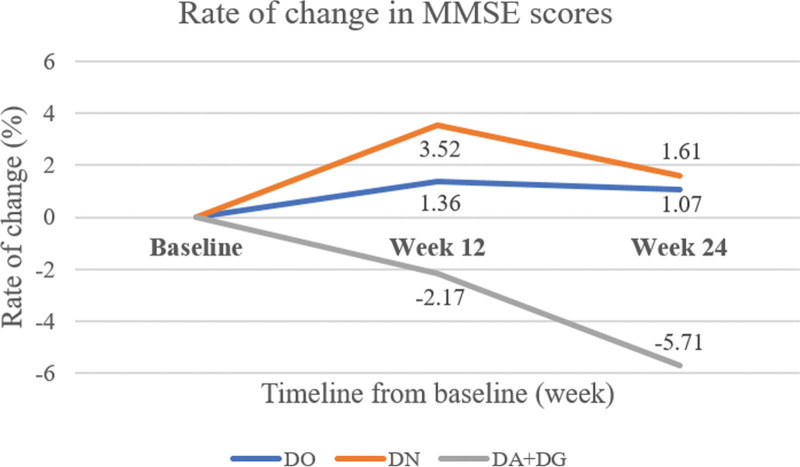
The rate of change in MMSE scores at the 12th week and the 24th week of study initiation. MMSE = mini-mental state examination.

The mean baseline MMSE scores of each group were summarized in Table 2. There was no significant difference among the groups. At the 12th week, it increased by 1.36% in the DO group, whereas it increased by 3.52% in the DN group. The MMSE score of the DA + DG group decreased by 2.17%. When comparing the results of the DO group with those of the DN group, there was a trend (*P* = .223). Comparison of the DN and DA + DG groups showed a significant difference (*P* = .026). At the 24th week, the MMSE score increased by 1.61% in the DN group (1.07% in the DO group, *P* = .771), while it decreased by 5.71% in the DA + DG group (*P* = .029). Figure 2 shows the rate of change in MMSE scores at the 12th week and the 24th week of study initiation, respectively.

**Table 2:** The initial MMSE scores at the start of the study for each group.

Group categorization	MMSE score (mean ± standard deviation)
Group 1 (DO)	26.02 ± 2.40
Group 2 (DN)	25.32 ± 3.11
Group 3 (DA) + group 4 (DG)	25.26 ± 3.48

DA = administration of donepezil and acetyl-l-carnitine, DG = administration of donepezil and ginkgo biloba extract, DN = administration of donepezil and choline alfoscerate together, DO = administration of donepezil alone.

### 3.3. Secondary endpoints

**Figure 3.**
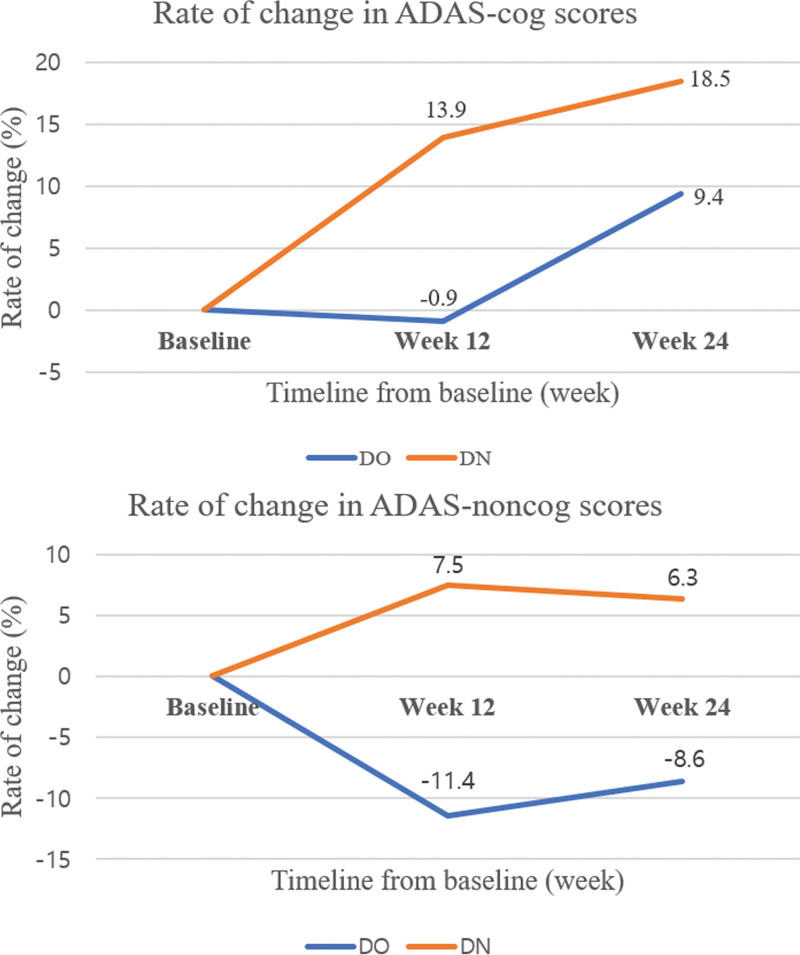
The rate of change in ADAS scores at the 12th week and the 24th week of study initiation. ADAS = Alzheimer disease assessment scale.

The ADAS-Cog score at the 12th week worsened by 0.9% in the DO group, whereas it improved by 13.9% in the DN group (*P* = .089). The ADAS-Noncog score at the same time point worsened by 11.4% in the DO group but improved by 7.5% in the DN group (*P* = .027). At the 24th week, the ADAS-Cog score improved by 9.4% in the DO group, and by 18.5% in the DN group (*P* = .290). The ADAS-Noncog score worsened by 8.6% in the DO group but improved by 6.3% in the DN group (*P* = .109). Figure 3 depicts the rate of change in ADAS scores at the 12th and the 24th weeks from the baseline.

### 3.4. Frequency analysis of cognitive parameters

Further additional analysis was attempted to determine the responders. The data were classified into improvement or worsening, and the frequency of improvement for each group was assessed at the 24th week. “Improvement” was defined as an increase or maintenance of the score, considering the clinical course of declining cognitive function in the natural neurodegenerative process.

**Figure 4.**
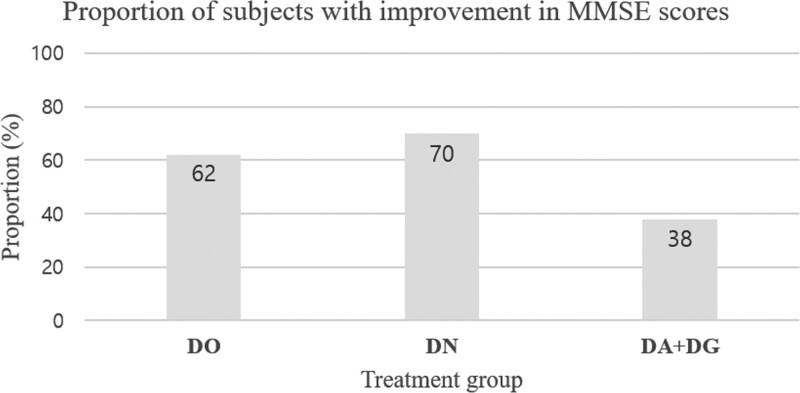
The frequency of improvement in MMSE scores for each group. MMSE = mini-mental state examination.

**Figure 5.**
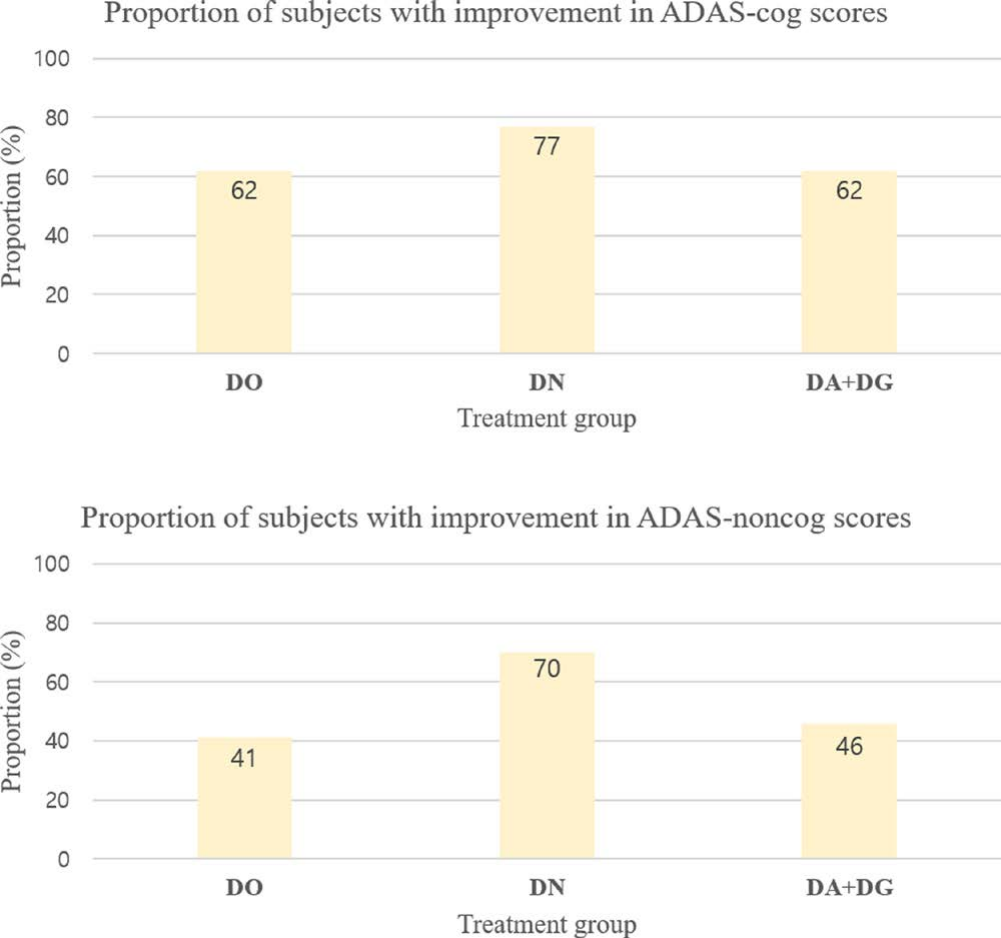
The frequency of improvement in ADAS-Cog and ADAS-Noncog scores for each group. ADAS-Cog = Alzheimer disease assessment scale-cognitive subscale, ADAS-Noncog = Alzheimer disease assessment scale-noncognitive subscale.

MMSE scores showed improvement in 62% of the subjects in the DO group, in 70% of the subjects in the DN group, and in 38% of the subjects in the DA + DG group. Figure 4 describes the frequency of improvement in MMSE scores for each group. For ADAS-Cog scores, improvement was also noted in 62% of the subjects in the DO group, in 77% of the subjects in the DN group, and in 62% of the subjects in the DA + DG group. For ADAS-Noncog scores, improvement was found in 41% of the subjects in the DO group, in 70% of the subjects in the DN group, and in 46% of the subjects in the DA + DG group. Figure 5 describes the frequency of improvement in ADAS-Cog and ADAS-Noncog scores for each group.

## 4. Discussion

According to the ASCOMALVA trial, the purpose of this study was to assess the reproducibility of the additional effect of choline alfoscerate in combination with donepezil, focusing on both cognitive and noncognitive domains. The results demonstrated that the combination of donepezil with choline alfoscerate was more effective in enhancing cognitive function compared to donepezil alone or donepezil with other nootropic agents. Consequently, these findings align with and support the results of the ASCOMALVA trial.

In this study, the design involved a mixed double-blind randomized controlled trial and an open-label observation trial. Originally, the plan was to randomize participants into 4 groups: DO, DN, DA, and DG. However, due to changes in the national insurance system, the use of acetyl-l-carnitine or ginkgo biloba extract as nootropics was limited. Consequently, the number of subjects in the DA or DG group was insufficient to allow for an equal comparison. As a result, further modification was made wherein a double-blind randomized controlled trial was applied to the DO and DN groups, while an open-label observation trial was applied to the DA + DG group. We consider the “DA + DG” group as another active and comparable control to the DN group. Our study demonstrated a more favorable trend with the DN group compared to the DO group. Additionally, the DN group exhibited superiority over the DA + DG group in both cognitive and noncognitive domains according to our study protocol. Several small-scale studies comparing the effects of choline alfoscerate with other cognition-enhancing agents such as CDP-choline, oxiracetam, and acetyl-l-carnitine in patients with neurodegenerative dementia and vascular dementia have also confirmed the superiority of choline alfoscerate.^[10]^ However, it is essential to interpret these findings carefully, as oxiracetam, acetyl-l-carnitine, or other nootropics should not be conclusively considered ineffective.

For instance, the timing of when donepezil was administered could be considered a confounding factor. Trials involving donepezil have shown more significant improvement during the first 1 to 2 years compared to the baseline. In both the DO group and the DN group, there were instances where donepezil had been newly added and maintained for at least 3 months if the MMSE score was 26 or less and the CDR score was 1.0 or less. Subsequently, these participants were randomized into either the DO or the DN group. Consequently, it was plausible that any further improvement in cognitive function could potentially be attributed to the effects of donepezil itself.

In our study, the DN group exhibited a 2.6-fold improvement at the 12th week and a 1.5-fold improvement at the 24 week in MMSE score compared to the baseline time point. The DO group also demonstrated improvement to a certain extent. However, in the DA + DG group, cognitive decline was observed over time. In many instances within the DA + DG group, the only way to assess the effectiveness of a drug was to observe the subjects who maintained on the drug. Consequently, in the DA + DG group, there was another possibility of a progressive clinical course, rather than choline alfoscerate superiority to other nootropics in our study.

Additionally, it appeared that there was a maximal cognitive enhancement time point within the 24 weeks. In our study, there were more improvements in the change rate at the 12th week, and this trend was sustained until the 24th week. It can be postulated that the speed of the progressive declining course and the speed of improvement in cognitive function by choline alfoscerate might be reached around these time points. Long-term follow-up, dose readjustment trials, or withdrawal may be warranted for maintaining strategies of choline alfoscerate and other nootropics.

ADAS-Cog, being more sensitive than MMSE, is reflective of cognitive function. Consequently, statistical significance may depend on the cognitive evaluation tools used. In this study, when their score changes were transformed into categorical data, similar patterns of responder rates were observed, suggesting that the significance can be interpreted by each cognitive evaluation tools.

ADAS-Noncog measures the noncognitive domain, including behavior. In dementia, behavioral abnormalities significantly interfere with daily life and particularly affect caregivers and people around them, making it most challenging to manage dementia patients. In this study, after using choline alfoscerate, an improvement of 18.9% was observed compared to the DO group at the 12th week, and an improvement of 14.9% was observed at the 24th week, supporting the ASCOMALVA trial.

## Acknowledgments

This research received grants from JW Pharmaceutical in Seoul, Korea.

## Author contributions

**Writing – original draft:** Wankiun Lee.

**Writing – review & editing:** Wankiun Lee.

**Conceptualization:** Manho Kim.

**Formal analysis:** Manho Kim.

**Funding acquisition:** Manho Kim.

**Supervision:** Manho Kim.
